# Current practice in the management of syncope in Italian hospitals: a national survey

**DOI:** 10.1093/europace/euag089

**Published:** 2026-05-13

**Authors:** Michele Brignole, Ivo Casagranda, Samuele Agusto, Marco Capacci, Giulia Matteucci, Giulia Rivasi, Andrea Ungar

**Affiliations:** Department of Cardiology, IRCCS Istituto Auxologico Italiano, Faint and Fall Research Centre, S. Luca Hospital, Piazzale Brescia 20, Milano 20149, Italy; Azienda Ospedaliero Universitaria “Santi Antonio e Biagio e Cesare Arrigo” Alessandria, Italy; Division of Geriatric and Intensive Care Medicine, University of Florence and Azienda Ospedaliero Universitaria Careggi, Florence, Italy; Division of Geriatric and Intensive Care Medicine, University of Florence and Azienda Ospedaliero Universitaria Careggi, Florence, Italy; Division of Geriatric and Intensive Care Medicine, University of Florence and Azienda Ospedaliero Universitaria Careggi, Florence, Italy; Division of Geriatric and Intensive Care Medicine, University of Florence and Azienda Ospedaliero Universitaria Careggi, Florence, Italy; Division of Geriatric and Intensive Care Medicine, University of Florence and Azienda Ospedaliero Universitaria Careggi, Florence, Italy

## Introduction

The European Heart Rhythm Association^1^ and the European Society of Cardiology^2^ recommend developing syncope facilities or syncope units (SU) and have outlined key management requirements. How these recommendations are applied in Europe remains unclear, lacking national surveys, with one exception.^3^ This paper reports the results of a national survey conducted in Italy in the year 2025 by the Italian Multidisciplinary Syncope Group (GIMSI).

## Method

**Figure 1:**
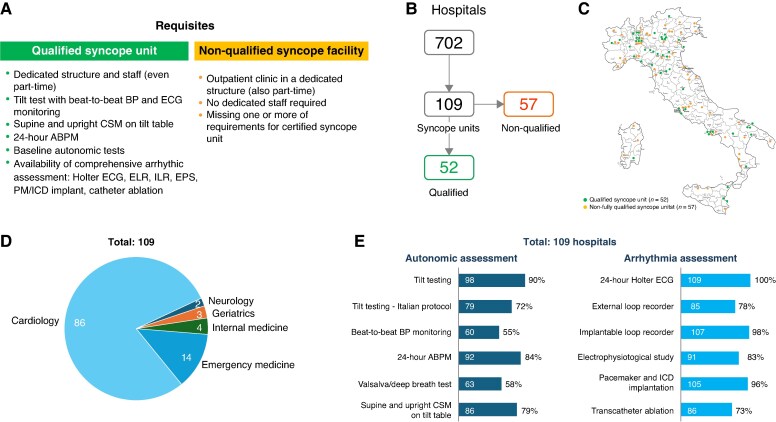
This figure summarizes the current practice in the management of syncope in Italian hospitals. Panel A. Definitions of syncope unit. Qualified syncope units were defined as those facilities that meet all the requirements of the best European standards. Those structures that were lacking one or more of these requirements were classified as not-fully qualified syncope units. Panel B. Main results of the survey. Panel C. Distribution of syncope facilities on the national territory. The 110 Italian provinces are shown, 44 of them showing no syncope unit. Panel D. Distribution according to specialties. Panel E. Number and percentage of hospitals where autonomic and arrhythmia assessment are available. In addition to essential requirements, the availability to perform baseline autonomic function tests (Valsalva manoeuvre, deep breathing test) and the availability, inside the hospital, of a complete arrhythmia assessment and therapy (electrophysiological study, pacemaker and ICD implantation, catheter ablation of arrhythmias) was also counted. *Abbreviations*: BP = blood pressure; ABPM = ambulatory blood pressure monitoring; CSM = carotid sinus massage.

All Italian hospitals were screened for the presence of any form of structured specialized SU between March and October 2025. An initial list comprising the 225 Local Health Authorities and the 104 academic hospitals within the Italian National Healthcare System was compiled using the official websites of Ministry of Health and of Italy's 20 regions. Subsequently, the hospitals affiliated with each Local Health Authority were identified from its official websites. In total, 702 Italian hospitals were identified, 571 of them with an emergency department. Each hospital's medical direction or public relations office was contacted by email or phone to determine whether a structured SU was operating there; if so, the name of the contact physician responsible of such structure was requested. If no information was found, a web search was conducted for public information about the hospital using the terms ‘syncope unit’ and ‘syncope clinic’ and the physician responsible of such structure was personally contacted by one of the authors. Finally, the directors of the 109 identified SU were requested to verify that their facilities fulfilled the following criteria:(i) the presence of an outpatient unit dedicated to the care of syncope patients, (ii) at least one physician with expertise in syncope, and (iii) formally appointed medical, nursing, and technical personnel. All of them signed and returned an appropriate questionnaire on resources and equipment of the SU, which is shown in the panel A of the *Figure 1*. Qualified SU were defined as those facilities that meet all the requirements of the best European standards.^1,2,4–6^ Those structures that were lacking one or more of these requirements were classified as not-fully qualified SU.

## Results

Overall, there were 109 hospitals (16%) with a SU, but only 52 (48%) of them met all the requisites for qualification (panel B of the *Figure 1*): 81% of them were in local hospitals and 19% in academic hospitals. The complete list of Italian SU can be found on www.gimsi.it. SU were present in all Italian regions although they showed a non-uniform distribution, being more often located in metropolitan areas and absent in 44/110 (40%) provinces into which it is subdivided the national territory (panel C of the *Figure 1*). Most SU (79%, *n* = 86) were in cardiology departments, the remaining being in emergency department (13%, #14), internal medicine (4%, #4), geriatric department (3%, #3) and neurology department (2%, #2), panel D of the *Figure 1*. Most facilities (95%, *n* = 104) were located in hospitals with emergency departments. Among them, 100 had formal referral procedures to the SU, and 72 conducted initial syncope evaluations within a 48-h stay in the Intensive Observation Unit. Availability of autonomic and arrhythmia tests is detailed in panel E of the *Figure 1*. The three most important autonomic tests necessary for the standardized two-step diagnostic work-up,^6^ i.e. tilt testing, supine and standing carotid sinus massage and 24-h ambulatory blood pressure monitoring, were available in 74 (68%) syncope facilities. Tilt testing was performed in 98 (90%), supine and standing carotid sinus massage was performed in 86 (79%) and 24-h ambulatory blood pressure monitoring in 92 (84%) facilities.

## Discussion

A minority of Italian hospitals has an SU and even fewer meet all the essential requirements established by European scientific societies.^1,2,4–6^ SU are present in all Italian regions although they show a non-uniform distribution, being more often located in metropolitan areas and absent in 40% of provinces. Most SU are in cardiology departments. This explains why, in general, arrhythmia assessment is more frequently available than autonomic assessment.

Non-fully qualified SU show highly heterogeneous set-up and equipment. The major weaknesses are the lack of beat-to-beat BP monitoring and impossibility of performing carotid sinus massage in the upright position on a tilt table. Also, 24-h ambulatory blood pressure monitoring is not available in some units. These weaknesses significantly impair the diagnostic yield, as recent data demonstrated that a complete autonomic assessment is necessary to identify the predominant haemodynamic mechanism of syncope and effectively prevent recurrences.^4–6^ Thus, most Italian general hospitals are well-equipped to investigate cardiac syncope, but there is limited awareness of the importance of diagnosing autonomic syncope, the most common type in the population.

The optimal number and placement of SU remain uncertain. In a study including 9 SU in Italy,^7^ the averaged population served by each unit was 220.000 inhabitants. The epidemiological data indicate that an incidence of 360 patients with syncope are referred for specialistic evaluation every 100 000 inhabitants per year.^8^ Based on the above data, GIMSI has estimated that one SU every 200 000–300 000 inhabitants or one SU per each Local Health Authority (266.000 inhabitants on average) would allow for optimal coverage of the national territory. This figure translates in a total number of 200–300 SU in Italy.

Few data are available from other countries to allow for a comparison. A survey has been recently performed in the Netherlands,^3^ where a network of 20 SU exists over a total of 72 hospitals (28%), 45% of them meeting a 19-item score which covers the requirements of European guidelines. On average, in the Netherlands there is a SU for every 900 000 inhabitants which compares with one every 550 000 inhabitants in Italy. Moreover, Dutch SU are equally distributed among cardiology and neurology departments.

A European survey^9^ revealed that many respondents did not have SU (88%) or dedicated management algorithms (44%) at their institutions. Carotid sinus massage, autonomic testing, and tilt-table testing were inconsistently used.

No other survey is available for the other European countries. This study advocates for extending European surveys to encompass all countries within Europe.

## Data Availability

The datasets generated during and/or analysed during the current study are available upon reasonable request.
